# Resistance to Hypomethylating Agents in Myelodysplastic Syndrome and Acute Myeloid Leukemia From Clinical Data and Molecular Mechanism

**DOI:** 10.3389/fonc.2021.706030

**Published:** 2021-09-28

**Authors:** Guangjie Zhao, Qian Wang, Shuang Li, Xiaoqin Wang

**Affiliations:** Department of Hematology, Huashan Hospital, Fudan University, Shanghai, China

**Keywords:** decitabine, azacitidine, hypomethylating agents, resistance, acute myeloid leukemia, myelodysplastic syndrome

## Abstract

The nucleoside analogs decitabine (5-AZA-dC) and azacitidine (5-AZA) have been developed as targeted therapies to reverse DNA methylation in different cancer types, and they significantly improve the survival of patients who are not suitable for traditional intensive chemotherapies or other treatment regimens. However, approximately 50% of patients have a response to hypomethylating agents (HMAs), and many patients have no response originally or in the process of treatment. Even though new combination regimens have been tested to overcome the resistance to 5-AZA-dC or 5-AZA, only a small proportion of patients benefited from these strategies, and the outcome was very poor. However, the mechanisms of the resistance remain unknown. Some studies only partially described management after failure and the mechanisms of resistance. Herein, we will review the clinical and molecular signatures of the HMA response, alternative treatment after failure, and the causes of resistance in hematological malignancies.

## Introduction

DNA methylation, which adds a methyl group to the cytosine base in the context of a CpG dinucleotide by DNA methyltransferases (DNMTs), is a crucial epigenetic modification that regulates gene expression in normal tissue development, aging, and disease (1, 2). In many cancers, DNA methylation in CpG islands by DNMTs, which is associated with gene repression, is closely related to disease pathogenesis and progression (3). The Ten eleven translocation (TET) family mediates the formation of unmethylated cytosines through passive demethylation during cell division or active demethylation *via* the base excision repair pathway (4). Given that DNA methylation is a reversible process, expressing silenced genes and reprograming cells to a normal-like state by inhibiting DNMTs may have treatment potentials, leading to the research and development of DNMT inhibitors for cancer treatment. Two DNMT inhibitors, 5-AZA and 5-AZA-dC have been successfully applied in the clinic (5).

Hypomethylating agents (HMAs) are the first-line regimen for treating patients with intermediate and high-risk myelodysplastic syndrome (MDS) and significantly improve the overall survival (OS) (6). Some studies reported that HMA treatment in MDS cannot prolong the OS, but it was associated with improvement in patient-reported quality of life (QOL) and reduced leukemia transformation (7, 8). The application of HMAs to elderly patients with acute myeloid leukemia (AML) who were ineligible for intensive chemotherapy conferred an overall or disease-free survival advantage (9). However, there were clinically significant differences in the achievement of red blood cell transfusion independence or survival between azacitidine- and decitabine-treated older AML patients (10). In other tumors, such as ovarian cancer, melanoma, prostate cancer, and relapsed or refractory diffuse large B-cell lymphoma, HMAs are mainly used in combination with standard chemotherapy to improve the activities of cytotoxic drugs (11–15).

The active tri-phosphorylated metabolite of 5-AZA-dCTP, which is catalyzed and formed by intracellular kinases, is directly incorporated into DNA. For 5-AZA, the majority of 5-AZA-CTP is incorporated into RNA, whereas the remaining part is converted to 5-AZA-dCTP by ribonucleotide reductase (RNR) and is incorporated into DNA during replication (16). Since the cytotoxic action of 5-AZA-dC and 5-AZA is a result of its incorporation into DNA, it is an S-phase-specific agent and produces much greater cell kill of long phase cells than of plateau-phase cells (17, 18). In the process of DNA methylation, DNMTs establish covalent bond with the cytosine ring and are subsequently released after methylation, while the reaction is blocked with azacytosine. The covalent protein adducts impair the function of DNA and trigger DNA damage signaling, resulting in the degradation of “trapped” DNMTs by the proteasomal pathway (19). DNMT1 (preferentially), DNMT3A, and DNMT3B mutation at the catalytic site are still sensitive to 5-AZA-dCTP-mediated degradation. This indicates that covalent bond formation between DNMTs and 5-AZA-dCTP-incorporated DNA may not be necessary for its degradation (20), and the alternative process is not known. 5-AZA-dC treatment also increases the level of E3 ligase, TNF receptor-associated factor 6 (TRAF6), leading to the ubiquitination of DNMTs and lysosome-dependent degradation (21).

Anticancer activities of HMAs are executed by inducing the expression of tumor-repressor genes, stimulating immune responses, and reducing oncogene expression and angiogenesis, resulting in cell differentiation and death as well as inhibition of cell proliferation and the stem cell niche (22). However, the overall response rate (ORR) in intermediate- and high- risk MDS patients was approximately 50% or less (23), and some of them lost response during treatment. HMA treatment had no benefit in patients with chronic lymphocytic leukemia and non-Hodgkin’s lymphoma (24). The causes of the inefficiency and responsiveness remain unknow. In this review, we will shed light on the signatures predicting response to HMAs, combination strategies to overcome resistance, and the mechanism of resistance.

## Predicting the Response to HMAs

### Clinical Parameters

To evaluate the clinical response of patients with myelodysplasia or AML receiving treatment, the International Working Group (IWG) published and revised standard response criteria, including alteration of the natural history of the disease, cytogenetic response, hematologic improvement (HI), and quality of life (QOL) (25, 26). Complete remission (CR), partial remission (PR), and HI are standard clinical parameters for predicting response. Bone marrow blasts > 15%, abnormal karyotypes, and previous treatment with low-dose cytosine arabinoside have been reported to be independent indicators for lower response rates in MDS patients treated with 5-AZA (27). However, adverse cytogenetics (intermediate and poor), including chromosome 7 deletion, were confirmed to show higher response rates (28, 29). The WHO classification, and IPSS risk category were close to patient survival, but the response rate could not be predicted. In contrast, platelets ≥ 100 × 10^^^9/L and WBC<3.0 × 10^^^9/L before treatment, and platelet count recovery by the second cycle of 5-AZA-dC treatment can be used as an early predictive marker of response (30–32).

### Mutation

A series of gene mutations drive clone expansion and malignant transformation (33) and are significantly prevalent in hematological disorders. Studies have shown that TET2 and DNMT3A mutations (29, 32, 34) are linked to improved response to HMAs in MDS and related disorders; P53 mutation also predicted 5-AZA-dC -induced complete remission in patients with MDS (35). In contrast, mutations in ASXL1, CBL, RAS and SF3B1 genes are not associated with the prediction of response to treatment (32). According to a study of 15 gene mutation analyses in CMML patients, no somatic mutations (SRSF2, TET2, ASXL1, NRAS, DNMT3A, RUNX1, U2AF1, TP53, JAK2, KIT, KRAS, SF3B1, EZH2, IDH1 and IDH2) were significantly correlated with response to 5-AZA-dC (36). FLT3-ITD mutation did not affect the overall response rate (ORR) in patients with AML (37). Notably, an unbiased framework on investigating the role of several mutations in predicting HMA resistance in MDS showed that EZH2 mutation predicted a lower response, while IDH1 mutation was linked to a higher response rate; seven different mutation combinations including ASXL1, NF1, EZH2, TET2, RUNX1, SRSF2 and BCOR predicted the resistance to HMAs (38, 39). Wu et al. reported the co-occurrence of RUNX1 and ASXL1 mutations that were associated with a poor response to HMAs (40), it was summarized in **Table 1**. This indicates that the HMA treatment response might be affected by two or more mutations.

**Table 1:** Gene mutation and HMA response.

Mutation status	Patients with mutation	Good Response correlation	Reference
TET2 DNMT3A IDH1/IDH2 TET2+/-DNMT3A ASXL1 CBL KRAS/NRAS SF3B1	17/92 MDS/MPN/AML 8/92 MDS/MPN/AML 7/92 MDS/MPN/AML 24/92 MDS/MPN/AML 24/92 MDS/MPN/AML 3/92 MDS/MPN/AML 2/92 MDS/MPN/AML 12/92 MDS/MPN/AML	Yes Yes Yes Yes No No No No	(32)
TET2 DNMT3A IDH1/IDH2 NPM1 DNMT3A+NPM1 FLT3-ITD FLT3-TKD CEBPA	8/46 AML 8/46 AML 7/46 AML 9/46 AML 5/46 AML 3/46 AML 1/46 AML 5/46 AML	No Yes No No Yes No / No	(34)
TET2 DNMT3A ASXL1 CEBPA TP53 U2AF2 RUNX1 SRSF2 ITIH3 WT1 GATA2 BCOR SETBP1 STAG2	13/109 MDS 17/109 MDS 16/109 MDS 0/109 AML 15/109 MDS 15/109 MDS 10/109 MDS 5/109 MDS 13/109 MDS 2/109 MDS 5/109 MDS 3/109 MDS 6/109 MDS 7/109 MDS	No No No / Yes No No No No No No No No No	(35)
TET2 DNMT3A IDH1 KRAS NRAS SF3B1 TP53 SRSF2 EZH2 KIT JAK2 U2AF1	17/40 CMML 5/40 CMML 1/40 CMML 1/40 CMML 8/40 CMML 1/40 CMML 3/40 CMML 21/40 CMML 1/40 CMML 2/40 CMML 2/40 CMML 4/40 CMML	No No / / No No No No / No No No	(36)
FLT3-ITD NPM1	7/34 AML 34/126	No No	(37)
TET2 DNMT3A IDH1 IDH2 ASXL1 CBL KRAS NRAS SF3B1 NPM1 CEBPA TP53 U2AF1 RUNX1 SRSF2 EZH2 KIT JAK2 WT1 GATA2 BCOR NF1 ZRSR2 EVT6 PRPF8 PRPN11	93/367 MDS 62/367 MDS 17/367 MDS 23/367 MDS 134/367 MDS 19/367 MDS 10/367 MDS 32/367 MDS 52/367 MDS 22/367 MDS 9/367 MDS 52/367 MDS 51/367 MDS 65/367 MDS 73/367 MDS 33/367 MDS 3/367 MDS 21/367 MDS 11/367 MDS 10/367 MDS 34/367 MDS 29/367 MDS 19/367 MDS 18/367 MDS 17/367 MDS 17/367 MDS	No No Yes No No No No No No No No No No No No No* No No No No No No No No No	(38)
IDH1/IDH2	7/35 AML	Yes	(39)
RUNX1+ASXL1	18/84 MDS	No*	(40)

Yes, good response relationship; No, no correlation. No*, poor response rate.

### DNA Methylation

A critical mechanism of HMAs in anticancer treatment is demethylation. More researchers have explored the role of DNA methylation or demethylation in predicting the HMA response. Global hypomethylation (predominantly in CpG islands and CpG island-associated regions) was correlated with the response in AML patients treated with low-dose 5-AZA. However, for some genes such as LINE1, HOXA5, P15, and H19, the alteration of DNA methylation after treatment cannot predict response (41, 42). K Raj reported that the baseline level of P15 methylation was much lower in responders than non-responders (43). However, one study suggested that gene methylation (ERα, NOR, CDH1, NPM2, OLIG2, CDH13, CDNK2B, PGRA, PDZ and RIL) at baseline did not correlate with clinical response to 5-AZA-dC, in which a significant correlation between reduced methylation after more than four cycles of treatment and clinical responses was observed (44). Owing to the identification of some epigenetic enzyme mutations, and given that concurrent hypermethylation and hypomethylation after treatment complicated the predictive factors of the HMA response, DNA methylation cannot be used as a marker to predict the response to HMAs (45). While some methylation regions were still able to potentially predict the response, 167 differentially methylated regions (DMRs) of DNA at baseline, which were preferentially located at distal regulatory regions, distinguished responders from non-responders in CMML patients (36).

### Gene Expression

Gene expression may be a predictive marker for HMA response. In AML patients treated with 5-AZA-dC, different gene expression patterns can be used to identify the 5-AZA-dC response. Genes such as SLC24A3, MUM1, TNFSF9, DBN1, ABAT, and DDX52 were highly expressed, manifesting the response to treatment; in contrast, overexpression of IFI44L, IFI27, PDK4, MX1, FAS, and ITGB2 were uncorrelated to HMAs (46). CXCL4, CXCL7, CJUN, and CMYB were highly expressed in non-responder patients with CMML (36, 47). Although these genes are related to the inflammatory pathway, the mechanism by which the altered expression of these genes affects HMA responsiveness is not clear.

As described previously, DNMTs are implicated in DNA methylation, the expression levels of DNMTs might be related to the 5-AZA-dC response. In breast cancer, patients with high DNMT3A and DNMT3B protein expression levels, and to a less extent, DNMT1, were more sensitive to 5-AZA-dC (21). The target of DNMTs, micro-29b, was highly expressed in responsive AML patients and could predict the response (48). Gene expression mutually affects DNA methylation. Loss of MLL5, a novel histone lysine methyltransferase, was associated with resistance to low- dose 5-AZA-dC, reduced global DNA methylation in promoter regions, and reduced DNA demethylation (49). Reduced methylation of phosphoinositide-phospholipase C beta1 in the promoter region and subsequent high mRNA expression after 5-AZA treatment predicted the responsiveness (50).

## Options After HMA Failure

Even though HMAs treatment showed prolonged survival in patients, the response was almost transient, many patients lost sensitivity within two years (51). After HMA failure, patients with MDS or AML receive traditional AML-like chemotherapies, which include low-dose cytarabine, the combination of cytarabine and daunorubicin, purine nucleoside analogs, or investigational treatment (inhibitors targeting PD-1, CTLA-4, Ras, BCL-2, IDH1 and IDH2 mutations, TLR-2, AXL, TGF-beta, spliceosome, NED88 activating enzyme), some of which also benefit from switching to another nucleoside analog (52–72). We have summarized this in **Table 2**. Novel forms of HMAs, which could be taken orally, could potentially change the routine of administration (73). Compared to intravenous administration, the oral form of cedazuridine/decitabine (ASTX727) produced similar decitabine exposure and efficacy and was recently approved by the FDA (74). However, there is still a lack of randomized controlled trials that compare the investigational drugs with and without HMA to a single use of HMA, and whether target therapies combined with HMA could improve the OS or ORR remains unknown. In addition, CD47, CD33/CD3 antibody, FLT3 inhibitors and autophagy inhibitors (ROC-325) are also being investigated for the treatment of naïve high-risk MDS/AML and refractory/relapsed AML (75–78), and autophagy inhibitors may be very promising treatment options for hematological malignancies in the future. The efficiency of these novel treatments in relapsed or refractory AML/MDS after HMA failure is unknown. We believe that the administration of these novel drugs should be based on the molecular characteristics of patients who relapse after HMA treatment.

**Table 2:** Different strategies for patients with HMA failure.

Treatment	Patients	Response	Median OS	Reference
DAC After AZA failure	36 CMML or MDS	3 marrow CR, 2 SD+HI-E,1 SD+HI-P, 1 SD+HI-E.	7.3	(52)
	14 MDS	ORR, 19.4%	6	(53)
	25 MDS/MPN	ORR, 28%. 3, CR, 1 HI.	5.9	(54)
	6 MDS (High risk)	No response, ORR, 0	8.9	(55)
	4 s-AML	No response, ORR, 0	7	(56)
	21 MDS	ORR, 3/4. 2 pCR, 1 HI.	17.8	(57)
		ORR, 19%, 1 mCR, 3 HI.		
AZA after DAC failure	10 MDS	ORR, 40%. 2 mCR,2 HI.	22	(57)
Chemotherapy				
intensive^a^	13 MDS	ORR, 31%	4.4	(55)
	35 MDS	ORR, 3/22	8.9	(58)
low dose^a^	32 MDS	ORR, 0/18	7.3	(58)
IA	10 AML, 10 MDS	2 CR, 1 mCR in AML	6^b^	(59)
		2 mCR in MDS	7^b^	
7+3	173 MDS, 30 AML	CR+iCR 39%, 63%	9.3, 8	(60)
IDAC	44 MDS, 12 AML	CR+iCR 64%, 25%	10.9, 6.9	
PNA	90 MDS, 17AML	CR+iCR 34%, 21%	12.9, 4.4	
Clofarabine	20 MDS	ORR 33%	7.8	(61)
HSCT	37 MDS	ORR, 13/19	19.5	(58)
	2 MDS	ORR, 2/2	6.9	(55)
	1 AML, 4 MDS	1 AML CR, 2 CR in MDS	14, 24^b^	(59)
	68 MDS/CMML	3 y RFS, 23%	not available	(62)
Lenalidomide	38 MDS	ORR,36.8%, 7 CR, 1 mCR, 3 PR, 3 HI-E	15.4	(63)
Lenalidomide+AZA	3 MDS	3 CR	5,7,7^b^	(64)
Vorinostat+cytarabine	40 MDS	ORR, 6/40. 2 CR, 2 CRi, 2 HI	9.1	(65)
SGI-110 (GDAC)	56 MDS/AML	ORR, 14.3%, 2 CR, 3 HI, 2 mCR, 1 PR	7.1	(66)
Bemcentinib (AXL inhibitor)	43 AML/MDS Recruiting	Phase II	Waiting	NCT 03824080
CPX-351	23 MDS (anticipated) Recruiting	Phase II	Waiting	NCT 03957876
	Recruiting	Phase I	Waiting	NCT 02019069
	Recruiting	Phase II	Waiting	NCT 03672539
	Recruiting	Phase I	Waiting	NCT 03896269
Enasidenib (IDH2 inhibitor)	Recruiting	Phase II	Waiting	NCT 03383575
AG-120 (IDH1 inhibitor)	Recruiting	Phase II	Waiting	NCT 03503409
OPN-305 (TLR-2 antibody)	51 low risk MDS	ORR, 50%	not available	(67)
Rigosertib	199 AML/MDS/CMML	ORR, 27%	8.2	(68)
Rigosertib	Recruiting	Phase III	Waiting	NCT 02562443
Rigosertib+AZA	17 MDS	ORR, 59%	not available	(69)
Nivolumab	15 MDS	ORR, 13%, 0 CR/PR	8	(70)
Ipilimumab	20 MDS	ORR, 35%, 3 CR/PR	8	
Nivolumab+				
Ipilimumab	7 MDS	ORR 39%	8.4	(71)
Durvalumab	Recruiting	Phase II	Waiting	NCT 02281084
Pembrolizumab	Recruiting	Phase I	Waiting	NCT 02936752
H3B-8800	Recruiting	Phase I	Waiting	NCT 02841540
Venetoclax+/-AZA	70 MDS (anticipated) Recruiting	Phase I	Waiting	NCT 02966782
Venetoclax	Recruiting	Phase I	Waiting	NCT 03404193
Pevonedistat+ AZA	71 MDS (anticipated) Recruiting	Phase 2	Waiting	NCT 03238248
Sotatercept	36 low-risk MDS	ORR, 58%	not available	(72)

CR, complete remission; PR, partial remission; iCR, incomplete remission; SD, stable disease; HI, hematological improvement; mCR, bone marrow complete remission; ORR, overall response rate; HSCT, hematopoietic stem cell transplantation; s-AML, second AML; IDAC, intermediate- to high-dose cytarabine; PNA, purine nucleoside analog based fludarabine, cladribine or clofarabine; 7 + 3, cytarabine plus daunorubicin; CPX-351, daunorubicin and cytarabine; H3B-8800, spliceosome inhibitor; PD-1 inhibitor, Nivolumab, Durvalumab, Pembrolizumab; CTLA-4 inhibitor, Ipilimumab; a, not indicated; b, complete remission duration time.

## Resistance To HMA

### Membrane Transporters

Hydrophilic drugs, such as 5-AZA and 5-AZA-dC, are taken up into the cells by human membrane nucleoside transporters (hNTs), which include equilibrate nucleoside transporters (hENT1, hENT2, hENT3, hENT4) and concentrative nucleoside transporters (hCNT1, hCNT2, hCNT3) (79). However, 5-AZA and 5-AZA-dC exhibited different nucleoside transportability profiles. All seven hNTs showed the ability to transport 5-AZA, hCNT3 showed the highest transportation efficiency, whereas hENT1 and hENT2 showed modest and hCNT1 and hCNT3 showed poor transportation of 5-AZA-dC (80). In fact, hENT1 mainly takes up 5-AZA-dC in a Na+ independent manner, which determines the activity of 5-AZA-dC in human HCT116 cancer cells (81). The expression level of these genes may determine the concentration and activity of 5-AZA and 5-AZA-dC in the cells. A study of 12 patients showed that in primary AML blast cells, hENT1 mRNA expression was highly abundant compared to hCNT1, hCNT3, hENT2 and hCNT2, and the transportation of 5-AZA also predominantly depended on the activity of hENT1 (82). To determine how transporters mediate resistance to 5-AZA-dC, Wu et al. measured the expression levels of hENT1 and hENT2 in 98 patients with MDS and found that patients responding to 5-AZA-dC displayed significantly higher hENT1 expression levels than non-responders, whereas hENT2 did not (83). However, Qin et al. reported that the gene expression of hENT1, hENT2, hCNT3 was not different between responders and non-responders in 14 MDS patients, and these genes expression were also comparable at diagnosis and relapse (84). We still do not know whether these patients who achieved a response initially and finally relapsed showed reduced expression of hENTs in a large population, and whether MDS/AML patients with AZA resistance had low expression levels of hNTs compared to responders. Notably, the development of lipid nanocapsules (LNCs) to encapsulate 5-AZA-dC showed high activity in 5-AZA-dC resistant and sensitive leukemia cells and could potentially bypass these transporters (85).

### Metabolism of HMA

**Figure 1:**
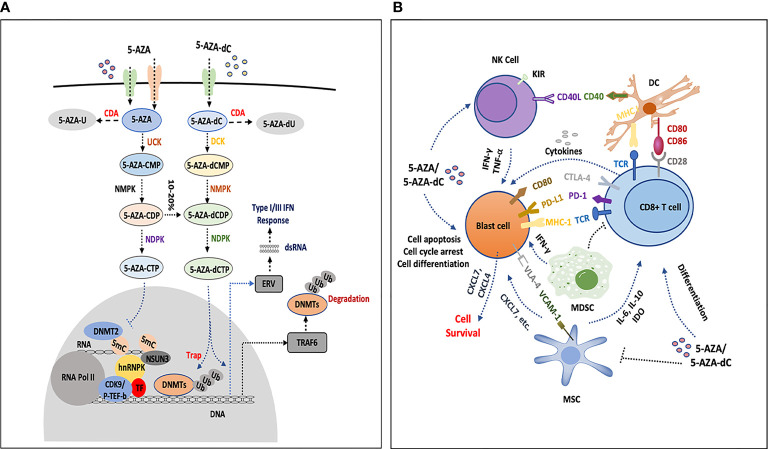
The influence of HMAs on bone marrow cells. **(A)** 5-AZA and 5-AZA-dC are transported into blast cells or immune cells (T cells, NK cells and DCs) by hENT1, hENT2, and hENT1-2, hCNT1 and hCNT3, respectively, part of which are catalyzed by CDA and result in degradation. The remaining AZA and 5-AZA-dC are catalyzed into 5-AZA-CMP and 5-AZA-dCMP by UCK and DCK, separately. In the following process, NMPK and NDPK play a role in the formation of 5-AZA-CTP and 5-AZA-dCTP. 5-AZA-CTP is mainly incorporated into RNA and possibly promotes the formation of 5-AZA sensitive chromatin structure, which consists of hnRNPK, DNMT2, NSUN3, CDK9/p-TEF-b and TF. 5-AZA-dCTP depletes DNMTs and increases gene expression, degradation of DNMTs is processed by proteosome pathway or by upregulating TRAF6 that leads to DNMTs ubiquitination, 5-AZA-dCTP is also activated and becomes hydrolyzed by SAMHD1 in leukemia. Simultaneously, 5-AZA-dCTP activates the transcription of ERV, induces dsRNA formation and facilitates type III interferon response. **(B)** 5-AZA-dC and 5-AZA inhibit NK cells by upregulating the inhibitory receptor KIR, or activate the antitumor activities by secreting some cytokines (IFN-Gamma or TNF-Alpha). HMAs induce the expression of CD40 and CD86 on DCs, which interact with CD8+ T cells by CD80/86-CD28 and TCR-MHC I CD8+ T cells communicate with blast cells by TCR-MHC I, PD-1-PDL1, and CTLA-4-CD80. HMAs increase the secretion of IFN-Gamma and antitumor activity of CD8+ T cells, and the expression of PD-1 and CTLA4 is upregulated as well, which participate in immune suppression. HMAs decrease the percentage of MDSCs in the bone marrow, which may promote the function of HMA. MSCs secrete IL-6, IL-10, IDO-1, etc., to inhibit CD8 T cells or directly interact with blast cells by VCAM1-VLA4. Leukemia cells express high level of CXCL4 and CXCL7 to promote the survival of leukemia cells under the treatment of HMAs. MSC can also secret CXCL7. These interactions and secretion of chemokines or cytokines in the bone marrow, which potentially mediate the resistance to HMAs, are still not completely understood.

After being transported into the cells, 5-AZA and 5-AZA-dC were catalyzed by a series of enzymes including deoxycytidine kinase (DCK), uridine cytidine kinase 2 (UCK2), cytidine deaminase (CDA), and carbamoyl-phosphate synthetase (CAD). 5-AZA-dC was converted to 5-AZA-dCTP and incorporated into DNA; however, most 5-AZA was incorporated into RNA as 5-AZA-CTP, and only 10%–20% is translated into 5-aza-dCTP after multistep catalyzation (21). Therefore, researchers believe that insufficient metabolites of 5-AZA and 5-AZA-dC might result in HMA resistance because of aberrant expression of metabolic genes. By measuring the expression of several genes encoding metabolic enzymes, Gu et al. found that mRNA expression of UCK2 and CDA increased in 5-AZA-dC treated MDS patients at relapse, while DCK expression was decreased compared to pre-treatment levels. In contrast, at relapse, DCK expression was upregulated while UCK2 and CDA expression was reduced. The expression of the *de novo* pyrimidine synthesis enzyme CAD increased in patients who resisted to both 5-AZA-dC and 5-AZA (86). In another study of 32 MDS patients who were either resistant or sensitized to 5-AZA-dC, DCK and CDA gene expression levels were comparable, but the ratio of CDA to DCK was significantly higher in non-responders than in responders, suggesting that this could be a mechanism of primary resistance (84). Mutations in genes that encode metabolic enzymes may affect HMA metabolism, and switching mutation of DCK from heterozygosity to homozygosity impaired the 5-AZA-dC sensitivity in the HL-60 cell line. However, no such mutation has been found in MDS patients (84, 87). In addition, the sterile alpha motif and histidine-aspartate domain-containing protein 1 (SAMHD1) is a 2’-deoxynucleoside-5’-triphosphate (dNTP) triphosphohydrolase that interacts with DAC-TP (not AZA-TP) and influences DAC efficacy in leukemia cells. SAMHD1 expression is inversely correlated with the clinical response to 5-AZA-dC in AML patients (88). We summarized them in the **Figure 1A**. This indicates that gene expression related to dNTP metabolism potentially interferes with 5-AZA or 5-AZA-dC activity and needs further investigation.

AZA incorporation may also be associated with 5-AZA sensitivity. By applying AZA mass spectrometry (AZA-MS) to primary bone marrow samples of MDS/CMML patients undergoing 5-AZA therapy, responders showed greater incorporation of 5-AZA-CdR into DNA than non-responders; however, much higher free AZA and AZA-RNA were observed in non-responders, which may have resulted from a shift in azacitidine/cytidine nucleotide ratios in the cytoplasm of non-responders. RNA methylation was not changed in either group of patients (89). In this study, it showed that the incorporation of 5-AZA-dCTP into DNA affected anticancer activity. Given the difference in the cell cycle, cell viability and gene expression between 5-AZA and 5-AZA-dC (90), the 5-AZA-CTP-RNA should have its unneglectable function and worth investigating.

### T Cells and Immune Response

Several groups have proposed that the clinical benefits for patients treated with HMA may be the result of direct cytotoxic and differential effects or immune responses for malignant cells. The role of HMA in the frequency and function of natural killer (NK) cells, T cells, and dendritic cells (DCs) has been reviewed previously (91). Overall, there is no consensus on how HMA affects the functionality of immune cells, and the role of DCs and NK cells in HMA responsiveness remains unknown. In this study, we mainly present the association of T cells with the HMA response, as briefly described in **Figure 1B**.

In a T-cell lymphoma mouse model, 5-AZA-dC stimulated CD80 expression in malignant cells and upgraded the cytolytic activity of IFN- γ- producing CD8+ T-cells (92). Low-dose 5-AZA-dC enhanced the activation and proliferation of human IFN-gamma+ T cells, as well as Th1 polarization and activity of cytotoxic T cells in solid tumor patients, increased IFN- γ+ T cells, and increased T-cell cytotoxicity predicted improved ORR and survival (93). In contrast to another study, Zhao et al. reported that 5-AZA-dC treatment was associated with increased expression of inhibitory receptors on T cells and reduced T cell population in elderly patients with AML. When comparing the differences in T cell differentiation and phenotypes between responders and non-responders, they found more naïve and central memory T cells, and inducible T cell costimulatory (ICOS)-expressing CD8+ T cells in responders, and found a specific immune signature predicted the response to 5-AZA-dC (94). These observations suggest IFN-γ+ T cells are implicated in the 5-AZA-dC response. Of note, 5-AZA-dC inhibited Gamma Delta T cell proliferation and cytotoxicity, and induced the expression of KIR2DL2/3 on Gamma Delta T cells, which were less toxic than negative cells (95), Suggesting that T cell activation was a double-edged sword and needed further analysis in HMA responders and non-responders.

Several studies have shown that 5-AZA upregulates the expression of tumor-specific antigens and cytotoxic T-lymphocytes. HMA induces the expression of inhibitory receptors on the surface, such as T cell programmed death-1 (PD-1), PD-L1, PDL-2 and CTLA-4 (96). Persistent expression and engagement of PD-1 and CTLA-4 results in T cell exhaustion and tumor immune evasion (97). The expression of these receptors was associated with resistance to 5-AZA-dC and 5-AZA treatment, and may be exploited by target therapies (96). Combination treatment with PD-1 antibody showed higher ORR than 5-AZA-dC or 5-AZA alone in older AML patients who were ineligible for intensive chemotherapy (98). HMA triggered the expression of endogenous retroviral (ERV) elements, and increased the transcription of double-stranded RNA, innate type I or III interferon response by the MDA5/MAVS/IRF7 pathway (99). However, the difference in ERV activation between responders and non-responders has not been explored in MDS/AML. In another study, when comparing the 5-AZA-dC responders and non-responders, there was no differential expression of PD-1 before and after treatment (94). In contrast to a report from Nahas et al. they showed guadecitabine (GDAC) negatively regulated inhibitory accessory cells by decreasing PD-1 expressing T cells and AML-mediated expansion of myeloid-derived suppressor cells (MDSCs) in mouse model, therapy with guadecitabine resulted in enhanced leukemia-specific immunity as well, as manifested by increased CD4+ and CD8+ cells expressing IFN-γ (100). These inconsistencies are likely due to different models *in vitro* or *in vivo*, functional assays, concentration administration, etc.

The 5-AZA not only induces a cytotoxic CD8+ T-cell response, but also stimulates a shift from cytotoxic to regulatory T cells with a functional phenotype in proinflammatory Th1 cells, indicating a potent inhibition of tumor -specific T cell immunity by 5-AZA (101, 102). Given the inhibitory and activating effects of HMA on T cells, further research is needed to explore its role in mediating HMA responsiveness.

### Bone Marrow-Derived Cells

Bone marrow-derived cells, mesenchymal stromal cells (MSCs) and myeloid-derived suppressor cells (MDSCs), are essential parts of the bone marrow microenvironment in regulating the immune response (103). In the myeloma microenvironment, 5-AZA-dC treatment inhibited tumor growth and enhanced T cell infiltration by depleting monocytic myeloid-derived suppressor cells (M-MDSCs) (104), which has also been shown in AML (100). Nevertheless, the percentage of M-MDSCs in non-responsive patients is uncertain. The phenotypes, transcriptome, and epigenomics of MSCs in MDS were significantly different from those of healthy donors, following 5-AZA treatment, and the gene expression pattern of MSCs from MDS patients with response was closely clustered with that of healthy donors. MSCs from patients who failed to respond to 5-AZA and could not be programmed by HMA were associated with rapid adverse disease transformation (105). MSCs play a role in immune suppression by secreting high levels of indoleamine 2, 3-dioxygenase (IDO-1), or other cytokines, which are generated by DCs and tumor cells as well. IDO-1 expression was associated with the failure of 5-AZA treatment through immunosuppression, which reduced the number of infiltrating CD8+ T cells and shortened the overall survival in high-risk MDS patients (106). IDO-1 inhibitors were investigated alone or combination with immune checkpoint inhibitors in the clinic (107). In addition, MSCs also secret CXCL7 that may promote the survival of cells (108), it was supported by the report that leukemia cells express high levels of CXCL4 and CXCL7 which is associated with the HMA non-responsiveness (36). The impact of MSCs on HMA responsiveness, the microenvironment in MDS/AML, and differential mediators secreted by MSCs that are implicated in HMA non-response remain uncertain.

### Hematopoietic Stem Progenitor Cell

Accumulating evidence suggests that leukemia stem cells (LSCs) are responsible for chemoresistance and leukemia relapse, as they can self-renew and to differentiate into the heterogeneous lineages of leukemia cells (109). Even though HMA substantially reduced the LSC-containing population in patients with CR/iCR, it cannot eradicate LSC, which will finally re-expand when relapse occurs. In non-responders, there was no significant reduction in the size of the LSC-containing population (110). In MDS patients with monosomy 7, the clonal involvement in dominant CD45RA+ progenitor populations was not reduced following the 5-AZA response, which indicated the resistance of this compartment (111). Using RNA sequencing performed on HSPC cells, Unnikrishnan et al. found that cell cycle arrest predicts resistance to 5-AZA, and with 5-AZA response, the inflammatory pathway was activated. Although 5-AZA did not completely eliminate dysplastic clones upon response, it changed the clonal contribution, which enabled previously dormant clones with a lower mutational burden (112). Therefore, targeting LSCs may potentially improve HMA efficiency and prevent disease relapse. The LSC targets CD44, CD47, CD33, CD96, TIM-3, and CD123 antibodies are undergoing investigation (113, 114); in particular, TIM-3 antibody, MBG-453, which targets LSCs and leukemia blast cells, is used to combinate with HMA, and has shown encouraging response and durability (115). This will facilitate its clinical application and further research on LSC target therapies.

## Conclusion

Clinical studies have shown that patients with intermediate-and high-risk MDS or elderly old AML receive HMA treatment preferentially. HMAs have also been widely used in low-risk MDS (116), in combination with other chemotherapies to enhance the activity of cytotoxic drugs in leukemia, lymphoma and other solid tumors (117–119), as maintenance therapy after allogeneic hematopoietic stem cell transplantation (allo-HSCT), and as part of conditioning regimens before allo-HSCT (120–122). Researchers have been exploring how to predict the response, cause, and outcome of resistance because of non-responsiveness in some patients. Clinical parameters, DNA methylation, gene expression signatures, and specific immune cell counterparts are promising markers for predicting response. MicroRNAs, such as microRNA-181 (123), microRNA-29c (124), microRNA-124 (125), and microRNA-29b (48) were also associated with HMA response. However, the role of long-noncoding RNAs (lncRNAs) in predicting HMA responsiveness and inducing the resistance is unknown.

New combination strategies have been developed to alleviate the resistance of HMAs and demonstrate the advantages of their safety and efficiency. It is important that more clinical trials are conducted to better understand of the mechanisms of resistance. Recently, Cheng et al. found 5-AZA-resistant MDS and AML patients showed a significant increase in RNA:m5C and NSUN1/BRD4-associated active chromatin. HnRNPK interacts with the lineage-determining transcription factors (TFs), GATA1, SPI1/PU.1, and CDK9/P-TEFb to recruit RNA-polymerase-II at nascent RNA, leading to the formation of an AZA-sensitive chromatin structure (**Figure 1A**) (126). Notably, 5-AZA inhibited cytosine 38 methylation of tRNA, a major substrate of DNMT2, resulting in tRNA hypomethylation (127). These studies suggested that 5-AZA was involved in RNA demethylation and that RNA demethylation affects the sensitivity to HMAs by modeling chromatin.

Importantly, 5-AZA-dC and 5-AZA induce cell cycle arrest at G1 phase *via* p21 and G2/M phase *via* p38 MAPK kinase pathway (128). However, some reports showed both drugs induced a G2/M-arrest in P39 and HL-60 leukemia cell lines, but not in KG-1 and MDS-1 cells (129). They both inhibit cell proliferation by increasing genes expression, such as Cylcin-D, p21, MyoD (130). In addition, the gene expression of HO-1 affects the efficacy of 5-AZA-dC by decreasing the expression of cell cycle related protein p15 (131). High expression of the melanocyte late-differentiation driver, SOX9, also upregulates the expression of cyclin-dependent kinase inhibitors (CDKN) p27/CDKN1B and p21/CDKN1A that mediate cell cycle exit with differentiation (132). When comparing the gene expression differences between MDS patients with HMA non-responsiveness and HMA responsiveness, Ashwin U, et al. found that cell cycle quiescence of hematopoietic progenitors marked AZA non-responders, targeting cell cycle quiescence might overcome AZA resistance (112). In CMML patients, HMA non-responders have high expression of CXCL4 and CXCL7, both of them are related to cell cycle activity (36). It indicates that cell cycle plays the essential role in HMA resistance and deserves further investigation.

The immune cells, T cells, NK cells, and DCs are essential components of the bone marrow microenvironment, which can be programmed by HMAs and participate in resistance, **Figure 1B**. The monocyte subset repartition after treatment is also a useful tool for predicting HMA response (133). These cells may antagonize the function of HMAs and promote leukemia cell survival by interacting with leukemia cells directly or indirectly secreting a variety of cytokines and chemokines, or in the opposite way. The functioning of these cells in the bone marrow to confer HMA resistance is not completely understood. Single-cell sequencing has the advantage of distinguishing novel cell populations and plotting gene expression patterns of different cell types, which will lay the foundation for exploring the mechanism of HMA resistance.

## Author Contributions

GZ and QW conceived and wrote the manuscript. GZ and SL did the figure and table. XW reviewed this manuscript. All authors contributed to the article and approved the submitted version.

## Funding

This project is founded by National Nature Science Foundation of China NSFC 81100389 and 82170136.

## Conflict of Interest

The authors declare that the research was conducted in the absence of any commercial or financial relationships that could be construed as a potential conflict of interest.

## Publisher’s Note

All claims expressed in this article are solely those of the authors and do not necessarily represent those of their affiliated organizations, or those of the publisher, the editors and the reviewers. Any product that may be evaluated in this article, or claim that may be made by its manufacturer, is not guaranteed or endorsed by the publisher.
